# Identification of Vaccinia Virus Inhibitors and Cellular Functions Necessary for Efficient Viral Replication by Screening Bioactives and FDA-Approved Drugs

**DOI:** 10.3390/vaccines8030401

**Published:** 2020-07-21

**Authors:** Chen Peng, Yanan Zhou, Shuai Cao, Anil Pant, Marlene L. Campos Guerrero, Peter McDonald, Anuradha Roy, Zhilong Yang

**Affiliations:** 1 Division of Biology, Kansas State University, Manhattan, KS 66506, USA; chen.peng@nih.gov (C.P.); yanan9112@gmail.com (Y.Z.); scao@ksu.edu (S.C.); anilpant@ksu.edu (A.P.); marlene.campos40@gmail.com (M.L.C.G.); 2 High Throughput Screening Laboratory, University of Kansas, Lawrence, KS 66045, USA; petemcd@ku.edu (P.M.); anuroy@ku.edu (A.R.)

**Keywords:** poxvirus, vaccinia virus, STAT3, Gaussia luciferase, high-throughput screening, small molecule inhibitor, bioactives, FDA-approved drugs, antivirals

## Abstract

Four decades after the eradication of smallpox, poxviruses continue to threaten the health of humans and other animals. Vaccinia virus (VACV) was used as the vaccine that successfully eradicated smallpox and is a prototypic member of the poxvirus family. Many cellular pathways play critical roles in productive poxvirus replication. These pathways provide opportunities to expand the arsenal of poxvirus antiviral development by targeting the cellular functions required for efficient poxvirus replication. In this study, we developed and optimized a secreted Gaussia luciferase-based, simplified assay procedure suitable for high throughput screening. Using this procedure, we screened a customized compound library that contained over 3200 bioactives and FDA (Food and Drug Administration)-approved chemicals, most having known cellular targets, for their inhibitory effects on VACV replication. We identified over 140 compounds that suppressed VACV replication. Many of these hits target cellular pathways previously reported to be required for efficient VACV replication, validating the effectiveness of our screening. Importantly, we also identified hits that target cellular functions with previously unknown roles in the VACV replication cycle. Among those in the latter category, we verified the antiviral role of several compounds targeting the janus kinase/signal transducer and activator of transcription-3 (JAK/STAT3) signaling pathway by showing that STAT3 inhibitors reduced VACV replication. Our findings identify pathways that are candidates for use in the prevention and treatment of poxvirus infections and additionally provide a foundation to investigate diverse cellular pathways for their roles in poxvirus replications.

## 1. Introduction

Smallpox, one of the deadliest diseases in human history, has claimed more human lives than all other infectious diseases combined. In the 20th century alone, more than 300 million deaths were attributed directly to smallpox [1]. Variola virus (VARV) is the causative agent of smallpox. Vaccinia virus (VACV) is a close relative of VARV, and it was the first vaccine used to eradicate smallpox [2]. However, it has been shown that the de novo assembly of infectious horsepox virus using laboratory-accessible equipment and chemically synthesized DNA is possible [3]. Concerns have been raised that a similar method could potentially be used to reconstitute VARV, which poses a public health threat because VARV from insecure stocks or assembled in a laboratory could be used as a bioweapon. Moreover, many other poxviruses continue to threaten human health in the post-smallpox era. For example, monkeypox virus, a zoonotic poxvirus genetically similar to VACV and VARV, is endemic in Central and Western Africa [4,5]. Monkeypox virus was introduced into the U.S. by exotic pet trade. The pathogen was then transmitted to humans and led to 37 confirmed cases in the U.S. in 2003 [6]. In addition, several travel-related exports of monkeypox virus have been reported in the United Kingdom, Singapore, and Israel in the past few years [7,8,9]. Molluscum contagiosum virus, another example of a human-specific poxvirus, causes a common infection throughout the world that is especially frequent in children, sexually active adults, and immunocompromised individuals [10]. Moreover, many other poxviruses, such as buffalopox and cowpox viruses in Asia and Europe, respectively, are still causing animal diseases and economic loss [11,12,13]. 

Though smallpox was eradicated nearly 40 years ago, the first antiviral compound to treat smallpox was only recently approved by the Food and Drug Administration (FDA) in 2018 [14,15]. The concerns for drug-resistance and adverse reactions demand the discovery of more antivirals with diverse targets [16]. Moreover, the continued surveillance and discovery of novel therapeutics for poxvirus infections are important for preventing emerging and re-emerging poxvirus pathogens other than VARV. In fact, the U.S. federal government is actively seeking additional drugs to treat smallpox and other poxvirus diseases [15,17,18]. In the past two decades, several antivirals for poxviruses have been identified, including CMX001, Tecovirimat, and CMLDBU6128 [17]. However, mutant viruses resistant to these drugs have also been identified, strongly incentivizing the identification of other new antivirals for poxvirus infections [17,18]. In addition to identifying drugs targeting viral components, an alternative approach is to discover compounds that target cellular functions required for efficient viral replication. Cellular function-targeted drugs have the potential to be broad-spectrum and are less likely to develop drug resistance [19].

In this study, we developed a Gaussia luciferase (Gluc)-based, all-in-one plate and an unbiased screening approach for compounds that can inhibit the replication of VACV. We screened a chemical library by including over 3200 compounds from Selleck bioactives and FDA-approved drugs that have known cellular targets. We identified over 140 positive hits that target various cellular processes. In addition to compounds targeting cellular functions that are unknown to be important for VACV replication such as janus kinase-signal transducer and activator of transcription-3 (JAK-STAT3) signaling, the hits also include numerous compounds targeting cellular functions that are known to be required for efficient VACV replication, thus validating the effectiveness of our screening system. Many of these positive hits reported here represent candidates for the development of cellular function-oriented poxvirus antivirals. 

## 2. Materials and Methods

### 2.1. Cells and Viruses

HeLa (ATCC CCL-2) and NHDF (normal human dermal fibroblast; ATCC, Manassas, VA, USA; PCS 201-012) cells were purchased from ATCC and maintained in Dulbecco’s Modified Eagle’s Medium (DMEM) supplemented with 2 mM L-glutamine, 100 units of penicillin, 100 µg of streptomycin, and 10% fetal bovine serum (FBS). BS-C-1 cells (ATCC CCL-26) were purchased from ATCC and grown in Eagle’s Minimum Essential Medium (EMEM) supplemented as described above. The recombinant VACV—Western Reserve virus expressing Gluc under viral late promoter was described elsewhere, and it was a gift from Dr. Bernard Moss [20]. Wildtype and recombinant viruses were amplified in BS-C-1 cells, and they were cushion- and gradient-purified prior to use, as described elsewhere [20]. 

### 2.2. Virus Infection

A cell culture medium from cells at a 90% confluency were removed and replaced with VACV-WR or vLGluc diluted in EMEM supplemented with 2.5% FBS. Cells were infected for one h at 37 °C. The inoculum was then discarded, and cells were washed 2× times with PBS and maintained in a fresh medium. After 24 or 48 h, viruses from each treatment were collected by freezing/thawing 3 times, or the Gluc was directly measured in the culture medium using a Glomax luminometer (Promega, Madison, WI, USA) with a Gaussia luciferase flash assay kit (ThermoFisher, Waltham, MA, USA). Virus titers were determined by plaque assay in BS-C-1 cells with viruses serially diluted in EMEM-2.5% FBS.

### 2.3. Gaussia Luciferase (Gluc) Reporter Screening Assay

All compounds were acoustically transferred to the assay plates using ECHO555 (Beckman Inc. Sykesville, MD, USA) at a final desired concentration. All plates included DMSO and cytosine arabinoside (AraC) controls. Cells were trypsinized, resuspended in a fresh medium, and counted before mixing with diluted reporter viruses to reach the desired multiplicity of infection (MOI). The cell–virus mixture was then plated in 96-well plates pre-coated with library drugs along with positive (AraC) and negative (DMSO) controls. The plating procedure was carried out using an automated microplate dispenser, and 50 µL of the virus–cell mixture (~5000 cells) were dispensed into each well. The plates were then incubated at 37 °C with 5% CO_2_. After 24 h, luciferase activities were measured using a Glomax luminometer with a Gaussia luciferase flash assay kit (Thermo Fisher 16158).

### 2.4. Cell Viability Assays

For high-throughput screening, HeLa cells were plated in 384-well microplates at 2000 cells/40 μL per well in DMEM containing 10% FBS. Media and vehicle control (DMSO) wells were included in each assay plate. After 24 and 48 h of incubation, cytotoxicity was measured on an Enspire plate reader (Perkin Elmer, Waltham, MA, USA) using the luminescence-based CellTiter-Glo reagent (Promega). The percent cytotoxicity was normalized to the DMSO controls.

A 3-(4,5-dimethylthiazol-2-yl)-2,5 diphenyl tetrazolium bromide (MTT) assay was performed using an MTT assay kit (Cayman Chemical, Ann Arbor, MI, USA, Cat#10009365) to measure the cytotoxicity of the chemical inhibitors on cells in a non-high-throughput setting. Briefly, NHDF cells in 96-well plates were treated with DMSO or chemical inhibitors dissolved in DMSO and incubated for 24 h. After incubation, 10 µL of MTT reagent were added to each well, and cells were incubated for another 3 h. Next, a 100 µL crystal resolving solution was added to each well, and absorbance at 595 nm (UV light) was measured for each well after 18 h of incubation at 37 °C.

### 2.5. Determination of the Effects of Compounds on Gluc Enzyme Reaction

To determine whether the compounds directly inhibited the Gaussia protein activity, we optimized a biochemical Gaussia luciferase assay counter-screen in 384-well plate format. The optimized reaction consisting of 5 nM purified Gaussia Luciferase enzyme (NanoLight Inc., Pinetop, AZ, USA) was incubated with the compounds for 10 min at room temperature before the addition of Gluc Glow Assay reagents (NanoLight Inc.). After 10 min of incubation, luminescence was measured using the Perkin Elmer Enspire, and percent activity was normalized to the negative and positive controls.

### 2.6. High-Throughput Screening Data Processing and Statistical Analysis

The raw luminescence data from the primary screening of 2038 bioactives and 1190 FDA-approved drug collections (Selleck Inc.) were used to calculate percent inhibition, which was normalized to the negative (DMSO) and positive (AraC) controls. The hits were identified as compounds that inhibited the luminescence to a greater extent than plate median plus three standard deviations. The average Z’ score of 0.6 ± 0.05 indicated a good separation of the positive and negative controls and the suitability of the assay for screening. The 1042 primary hits were reconfirmed in a dose response at concentrations of 20, 10, 5, and 0 mM in anti-viral assays at both low and high MOIs, and they were also evaluated at 24 and 48 h for HeLa cytotoxicity and in the counter-screen for Gaussia reporter inhibitors. A final set of 142 compounds that showed potent anti-viral activity and were not cytotoxic or Gaussia reporter inhibitors were further analyzed by cheminformatics for their modes of action. 

## 3. Results

### 3.1. Development and Optimization of a Secreted Gluc-Based Assay Procedure to Screen VACV Inhibitors

**Figure 1:**
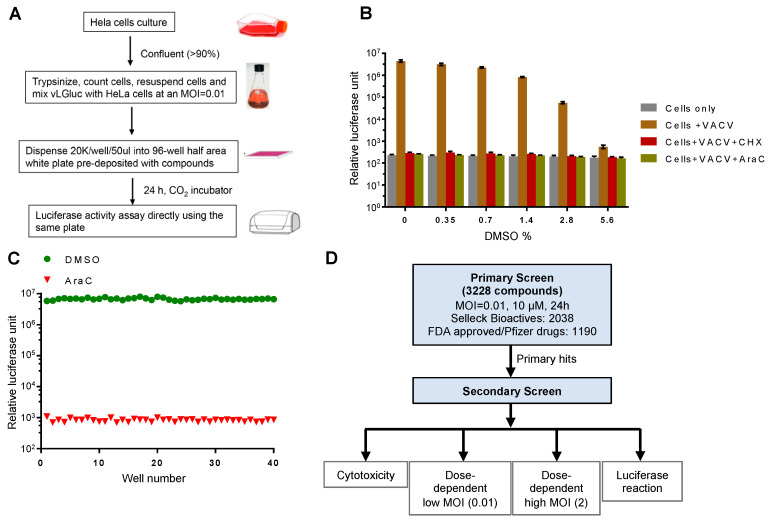
Experimental design and optimization of the screening procedure. (**A**) Experimental procedure for the luciferase reporter assay. (**B**) Effect of DMSO on the reporter assay. HeLa cells were infected with vLGluc at 0.01 PFU/cell in the presence or absence of cycloheximide (CHX) or cytosine arabinoside (AraC). DMSO was added to each well to reach the indicated final concentrations (*v*/*v*). Luciferase activities were measured 24 hours post infection (hpi). Error bars indicate standard deviation (n = 3). (**C**) HeLa cells from 40 wells in a 96-well plate at 90% confluency were infected with vLGluc at 0.01 PFU/cell in the presence of only DMSO or AraC diluted in DMSO. Luciferase activities were determined at 24 hpi. (**D**) Flowchart illustrating two rounds of screenings performed. MOI: multiplicity of infection.

We posit that Gluc is an outstanding reporter gene for a high-throughput chemical screen. It was released into the cell culture medium immediately after synthesis, which allowed it to be detected in real-time without the need to lyse the cells. Therefore, we used a reporter VACV, vLGluc, which was engineered to express Gluc in the WR strain of VACV under the control of a well-characterized viral late F17 promoter [20]. The luciferase expressed from this virus was found predominantly (99.8%) in the cell-culture medium. After extensive optimization and testing, we established a protocol for the screening, as illustrated in Figure 1A. Briefly, HeLa cells grown to >90% confluency were trypsinized, counted, and mixed with vLGluc at 0.01 PFU per cell. An automated dispenser was then used to dispense the virus–cell mixture into 96-well plates (50 µL/well), and the wells were pre-deposited with chemicals (one compound/well with desired concentration). The infection was incubated for 24 h prior to luciferase detection with a luminometer that directly injected detection reagent into the wells.

A screening protocol adaptable for high-throughput screening was optimized. First, in order to simplify the screening procedure as a way to reduce system variations and errors, we made changes to the typical VACV infection protocol of adherent cells by mixing cell suspension and viruses prior to plating cells in multi-well plates containing controls and compounds. Second, the Gaussia luciferase detection reagent was directly injected into the wells with infected cells at 24 h post infection (hpi) to eliminate variability resulting from media transfer to new plates. Third, we used a low MOI, which ensured the discovery of chemicals working on all steps of VACV replication and spread. Finally, the highly sensitive glow type Gluc signal was quite stable for detection. All steps were amenable to automated liquid dispensing. In this procedure, to counteract discrepancies caused by plate-to-plate variation, we included negative control wells containing DMSO only, which was used to dissolve drugs. We also included positive control wells with AraC, a well-established chemical to block VACV DNA replication and downstream post-replicative gene expression [21]. To test the effect of DMSO on Gluc expression from the virus, we performed a dose-dependent experiment using various DMSO concentrations. We found a minimal influence of DMSO at the concentration we used (<0.35%) for the screening (Figure 1B). Moreover, we optimized cell density (90% confluency), medium volume (50 µL/well), MOI (0.01), and incubation time (24 h) to increase the reproducibility of the assay. As a result, we were able to establish a very low well-to-well variation using AraC in the pilot test (Figure 1C).

Next, we assembled a customized drug library that contains 3228 compounds. This library includes drugs from the Selleck Bioactives and FDA-approved chemicals. One advantage of this library is that most of these compounds have at least one known cellular target, which would help identify new cellular processes/pathways utilized by VACV to facilitate its own replication. The library also provides redundancy because multiple compounds often target a common cellular target. The library was screened for the drugs’ inhibition on the expression of the Gluc reporter gene in a procedure illustrated in Figure 1D. For the primary screen, we screened the drugs with a low MOI to monitor their effect on spread using the procedure shown in Figure 1A. The positive hits were selected based on their ability to reduce luciferase production as compared to AraC (100%). For the secondary screen, we tested the primary hits using different doses of chemicals (5, 10, and 20 µM) at both low and higher MOIs (0.01 and 2, respectively). We also tested the cytotoxicity of the chemicals on HeLa cells and their direct effects on purified Gluc enzyme activity/reaction to exclude false positives (Figure 1D).

### 3.2. Identification of VACV Inhibitors Targeting a Broad Range of Cellular Targets and Processes

**Figure 2:**
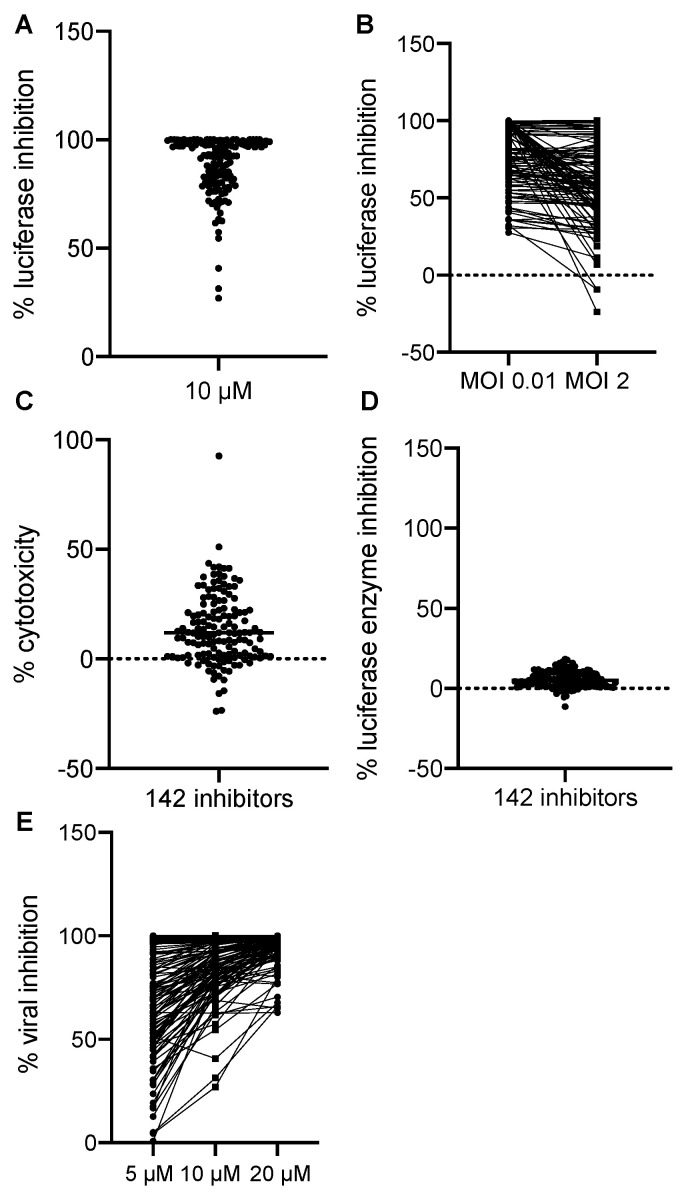
Screening results. (**A**) The effects of 142 positive hits at 10 µM were plotted. Each dot represents one compound. Luciferase activity from each well was normalized to that of the positive control wells containing AraC (100%) diluted in DMSO. (**B**) The inhibitory effect of all 142 positive hits at low (0.01) and high (2) MOIs were plotted. The % luciferase inhibition was calculated as described in (**A**). (**C**) Cytotoxic effects of all 142 positive hits were examined. DMSO’s effect was set to 0%, and the data of all hits were normalized to DMSO only. (**D**) The effect of positive hits on the luciferase reaction itself was examined by incubating the compounds with a purified Gaussia luciferase enzyme, and luciferase activity was measured after 10 min of incubation. Data from DMSO-only wells were set to 0%. (**E**) Dose-dependent effects of 142 hits were plotted. Data were acquired and normalized as described above.

We normalized all hits to the negative and positive controls where DMSO treatment alone was set to 0% and AraC treatment was set to 100% luciferase inhibition. All hits were plotted by their effects on Gluc production in the primary screen at 10 µM in Figure 2A. We found that most of the hits showed the inhibition of VACV replication at MOIs of 0.01 and 2. Some of the inhibitors had more potent effects of reducing VACV replication at the MOI of 0.01 (Figure 2B), thus suggesting that cell-to-cell spread was also affected by these hits. The majority of the hits showed a low cytotoxicity compared to DMSO, the solvent used for all drugs (Figure 2C). In addition, the positive effects we observed were not a result of the direct inhibition of the enzymatic activity or reaction of the Gluc (Figure 2D). To further characterize the inhibitors, we performed a dose-dependent study in which we included two more dosages (5 and 20 µM) in addition to the initial dose (10 µM). We found that most of them showed a dose-dependent inhibition of VACV replication, indicating a specific effect of the chemicals on VACV replication (Figure 2E). Overall, after the procedure, we identified 142 hits that significantly reduced Gluc activities in the viral reporter assay.

All 142 positive hits and their cellular targets are listed and clustered by their common cellular functions in Table 1. The most apparent category was nucleic acid analogs, which were predicted to have antiviral functions because they could be integrated into actively replicating DNA and could terminate DNA synthesis. The nucleic acid analogs would usually block cellular DNA replication in a similar mechanism; however, at the tested concentrations, the analogs showed no or low toxic effects on cell viability. The most abundant positive hits were found in the categories of HSP90 (heat shock protein 90) and EGFR (epidermal growth factor receptor) inhibitors, both of which have been previously shown to be required for efficient VACV replication [22,23]. In addition, microtubules, mTOR (mammalian target of rapamycin), proteasome, and CRM1 (chromosomal maintenance 1) have also been shown to be indispensable for VACV replication in cells by other techniques [24,25,26,27,28,29,30]. The fact that we were able to pick up many positive hits in these pathways known to be crucial for VACV replication demonstrated our screening method’s high effectiveness on identifying bona fide VACV inhibitors.

Remarkably, we also identified many hits that target biological processes that were previously unknown to promote VACV replication. Examples of those include inhibitors of the JAK-STAT pathway, topoisomerase II, and SIRT1 (sirtuin 1). The identification of these chemicals opens exciting new directions for understanding their roles in facilitating VACV replication and additional cellular targets for the development of new antivirals.

Interestingly, 35 of the identified positive hits were drugs already approved by the FDA for antiviral or non-antiviral-purposes. For example, floxuridine was approved in the 1970s for cancer treatment, and it belongs to the class of antimetabolites [67]. In addition, anagrelide is a drug used to treat essential thrombocytosis and chronic myeloid leukemia. It works by inhibiting the maturation of platelets from megakaryocytes [68]. Moreover, desmethyl erlotinib is an EGFR inhibitor, and it was approved in 2004 to treat lung and pancreatic cancer [69]. All FDA-approved drugs identified have some known modes of actions and have shown the potent inhibition of VACV in the screens (Table 1), suggesting a promising direction of exploring novel and safe antivirals for poxvirus infection.

### 3.3. Verification of the Suppressing Effects of JAK-STAT3 Pathway Inhibitors on VACV Replication

Among the positive hits, we noticed multiple compounds with a known function to inhibit the JAK-STAT pathway. The JAK-STAT signaling pathway is known to participate in various cellular processes such as immunity, cell division, cell death, and tumorigenesis [70]. The pathway is comprised of three major parts: receptors that could react to stimulations from different ligands under different physiological conditions, JAKs, and STAT proteins [70]. The disruption of the JAK-STAT pathway leads to a variety of diseases including cancer, immune disorders, and skin conditions [71]. In humans, there are four JAKs (JAK1, JAK2, JAK3, and TYK2) and seven STATs (STAT1, STAT2, STAT3, STAT4, STAT5A, STAT5B, and STAT6). The combination of different JAKs and STATs could result in the activation and/or inactivation of different cellular processes through transcriptional regulation [71,72]. The specific targets of the identified positive hits are listed in Table 2. Interestingly, among all JAK-STAT inhibitors, STAT3 was a convergent target found in our screening.

**Figure 3:**
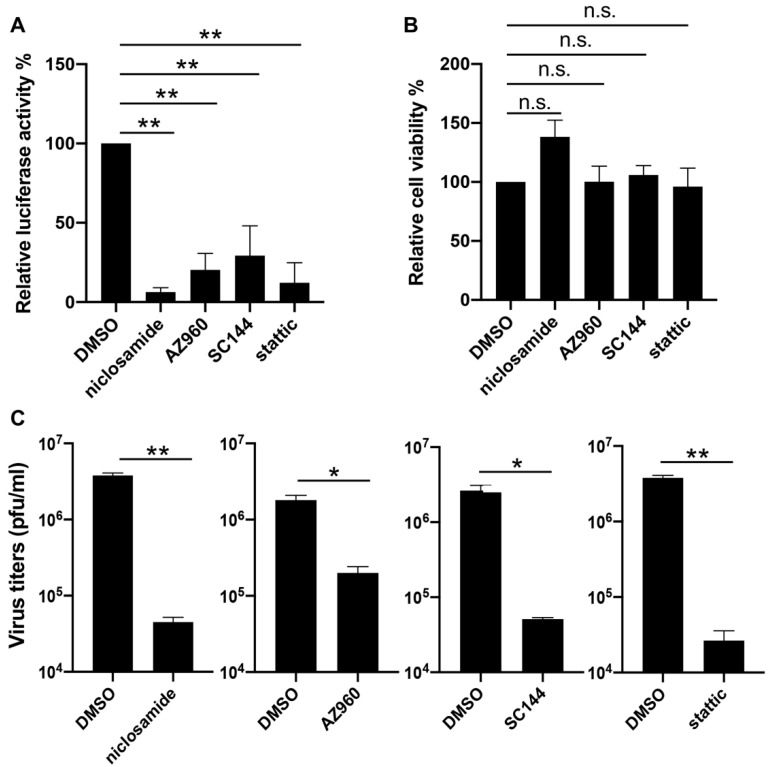
Inhibition of VACV by STAT3 inhibitors. (**A**) Normal human dermal fibroblast (NHDF) cells were treated with DMSO, niclosamide (1 µM), AZ960 (3 µM), SC144 (1 µM), or static (3 µM), and then they were infected with vLGluc at 1 PFU/cell. At 8 h later, luciferase activity was determined in collected cell culture media. (**B**) NHDF cells were treated with the above drugs for 24 h and cell viability was measured by an MTT (3-(4,5-dimethylthiazol-2-yl)-2,5 diphenyl tetrazolium bromide) assay. Three biological replicates were carried out. Error bars indicated standard deviation. (**C**) NHDF cells were treated with selected STAT3 inhibitors at the concentrations mentioned above and infected with VACV-WR at 0.01 PFU/cell. Viruses were collected at 48 hpi and tittered in BS-C-1 cells. Error bars indicate standard deviation (n = 3). Statistics: * *p* < 0.05, ** *p* < 0.01, n.s.: non-significant by two-sided Students’ T test.

To verify the effects of inhibitors on the JAK-STAT3 pathway in the VACV life cycle, we decided to first repeat the reporter assay with three compounds identified from the screen including SC144, AZ960, and niclosamide, each of which targets one component of the pathway, namely the ligand-receptor (SC144), the JAK2 kinase (AZ960), and the STAT3 protein (niclosamide). Additionally, we included a widely reported and selective STAT3 inhibitor static that was not picked up in our screening. We also chose to test the inhibitors in a different cell type: NHDF, which is a line of human dermal fibroblasts of non-cancerous origin. After optimizing the working concentrations for the drugs in NHDF cells (niclosamide and SC144: 1 µM, stattic: 3 µM), we determined late gene expression by infecting the cells with the same reporter virus (vLGluc) used in the drug treatment screenings, and we monitored luciferase activities at 8 hpi. Our analysis showed that all drugs significantly suppressed viral late gene expression without negatively affecting cell viability at the indicated concentrations (Figure 3A,B). Next, we sought to examine if blocking STAT3 with chemical inhibitors could directly impair viral replication. NHDF cells were treated with DMSO, niclosamide, AZ960, SC144, or stattic for one hour before they were infected with VACV-WR at 0.01 PFU/cell. Viruses were harvested at 48 hpi, and virus titers were determined by a plaque assay in BS-C-1 cells. As shown in Figure 3C, all four tested STAT3 inhibitors substantially repressed VACV replication, indicating that blocking STAT3 diminished VACV replication in human cells.

## 4. Discussion

After a series of optimizations, including adjustments to parameters such as cell number, infection time, and infection MOI, we developed an assay to screen anti-VACV compounds with all steps performed in one single plate. We screened a library with over 3200 compounds by performing multiple rounds of screening for their effects on virus spread, cell toxicity, and on the reporter assay itself at various doses, and we identified more than 140 positive hits that showed the statistically significant inhibition of VACV spread but manageable levels of toxicity on cell viability. The assay developed here could be readily scaled up for screening with a much higher capacity.

The utility and effectiveness of this assay were validated by the discovery of positive hits that targeted cellular pathways that were reported to be important for VACV replication. For example, Hung et al. reported that HSP90 redistributed upon VACV infection and a chemical geldanamycin (GA), which blocks the ATPase activity of HSP90, strongly suppressed virus infection in RK13 cells [22]. Moreover, Kowalczyk and coworkers observed that both HSP70 and HSP90 translocated to the nucleus at the early time of VACV infection, and the mRNA abundance of HSP90 was perturbed by the virus infection [73]. These results suggest that HSP90 may promote VACV replication. We found 12 different compounds in our screen that were previously known to inhibit HSP90, which further emphasized the importance of HSP90 in supporting VACV infection and encouraged more studies at the molecular level. It is possible that HSP90 assists the folding of VACV virion proteins for proper virus assembly. However, the actual mechanism needs more thorough investigations. Another example is the mTOR-related pathways. The mTOR is a key regulator of many cellular pathways that can respond to both extracellular and intracellular stimuli [74]. Many viruses have evolved strategies to promote, reduce, or even inhibit the mTOR pathway for their own replication [75]. It has been shown that VACV activates the mTOR pathway to delay apoptotic signals, which promote viral replication in mammalian cells [25]. However, it was also shown that the F17 protein made late during a VACV infection was able to dysregulate the activity of mTOR, which paralyzed its role in activating STING (stimulator of interferon genes) and the downstream antiviral responses [25]. In our screen, we found seven inhibitors of the PI3K/mTOR pathway that showed the significant suppression of VACV replication. Again, these findings demonstrated the effectiveness of our screening assay.

Importantly, we identified many compounds that target cellular functions that have not been previously reported as necessary for VACV replication including aryl hydrocarbon receptor (AhR), cytochrome P450, and aurora kinases A/B. Many of these pathways have been extensively studied in other fields, and tools are readily available for the manipulation for some of them. However, their involvement in poxvirus infections has not been previously reported. Admittedly, viruses are obligate intracellular parasites, and their reproduction heavily depends on the host cell’s machinery and metabolism. It is expected that the disruption of many essential cellular functions by chemicals or genetic alterations could lead to the arrest of cellular functions that will inevitably jeopardize virus replication. However, it is worth noting that all the positive hits reported here were tested for their effect on cell viability for up to 48 h at various concentrations and at concentrations where they exhibit the vigorous inhibition of virus gene expression, and most of them showed no or very low negative effects on cell viability. A possible explanation is that the virus might be capable of temporarily ramping up some cellular pathways to degrees that are only beneficial to the virus but superfluous to the cells. The chemical inhibition of those pathways would pose a more significant effect on viral replication than on cell metabolism. These pathways are, therefore, outstanding candidates for targeted antiviral therapy using chemical or genetic approaches. Our screening has provided multiple candidates for future research and validation, especially the thirty-five FDA-approved drugs.

We performed further analyses with inhibitors of the JAK-STAT3 pathway in primary cells. Our results confirmed that the inhibition of components diminished virus replication and late gene expression, suggesting a role of STAT3 in promoting VACV replication in human cells. Interestingly, another study published several years ago investigated the function of STAT3 in VACV-ACAM2000 infection, and the results suggested a defensive role of STAT3 in VACV infection [76]. It is worth noting that the studies differed in two key aspects, which may explain the observed differences. First, while the previous study was performed in human and mouse keratinocytes, we executed our screens in HeLa cells and confirmed our findings in NHDFs. Secondly, the prior study used the ACAM2000 strain of VACV, a clone derived from Dryvax [77]. ACAM2000 is a live attenuated strain approved as a smallpox vaccine [78]. VACV-WR, the virus used in our study, is, on the other hand, one of the most virulent VACV strains tested in animal models and was considered unsuitable to be used as a vaccine due to its high neuropathogenicity in mice models [79]. A comparison between the genomes of VACV-WR and ACAM2000 showed the ACAM2000 has two major deletions in the inverted terminal repeats (ITR, C12-C18 at the left ITR and the corresponding duplicates at the right ITR), as well as mutations throughout the genome [77,80]. Among those deleted, C12 has been shown to be important for enabling modified vaccinia virus Ankara (MVA) replication in human cells [81,82]. Some of those genes lost in ACAM2000 might regulate innate immune responses in cells, which complicates the role of STAT3 in VACV replication. Further investigations are needed to define the role of STAT3 in the replication of different VACV strains and elucidate the mechanism involved in the requirement of STAT3 signaling VACV replication.

Antivirals are indispensable therapeutics in preparing for emerging and re-emerging infectious diseases. They could be used post-virus exposure in patients and could work rapidly in constraining virus dissemination compared to vaccines. Our study has expanded the pool of therapeutic candidates for future pre-clinical and clinical tests to treat poxvirus infections. Further preclinical screening of the candidates listed in this study will be needed to evaluate drug effects in animal models, pharmacokinetics, the development of drug resistance, and in vivo toxicity before the most likely effective antiviral compounds for further clinical evaluation can be identified. Additionally and importantly, we identified new cell targets for understanding their roles in poxvirus infections and as targets for new antiviral development, which is complementary to a few previous genetic screens of cellular genes needed for efficient VACV replication [29,30,83].

## 5. Conclusions

Using a Gaussia luciferase-based reporter assay suitable for high-throughput screening, we identified over 140 compounds that could suppress VACV replication and spread in cultured human cells. These compounds are from a chemical library containing bioactives and FDA-approved drugs with known cellular targets. These cellular functions targeted by the positive hits include those previously known to be important for VACV replication, as well as those with previously unknown roles in supporting VACV replication. Further, we verified the inhibitory effects of multiple JAK-STAT3 inhibitors on viral replication. Our results provide promising candidate compounds for future evaluation in treating orthopoxvirus infections. The cell functions targeted by these compounds need additional investigations for understanding their roles in poxvirus infections. 

## Figures and Tables

**Table 1:** Vaccinia virus (VACV) inhibitors identified after two-round of screens. FDA: Food and Drug Administration.

Compound Name	Approved by the FDA (Y/N)	Primary Screen (% of Inhibition)	Secondary Screen (% of Inhibition)		Cytotoxicity Effect (% of Reduction)		Direct Gluc Effect (% of Reduction)	Cellular Target	Cellular Target Known to Interact with VACV?
		MOI 0.01	MOI 0.01	MOI 2	24 h	48 h	2 h		
StemRegenin 1	N	67.1	68.1	55.6	3.3	2.0	−8.4	AhR: aryl hydrocarbon receptor	No
Niclosamide (Niclocide)	Y	94.9	43.7	32.7	20.4	42.0	−6.4	Signal transducer and activator of transcription-3 (STAT3)	No
ICG-001	N	76.2	80.9	78.4	29.4	35.9	5.3	Wnt/β-catenin/TCF-mediated transcription a	[31]
Nifuroxazide	N	78.5	48.8	42.0	20.7	18.5	−5.9	STAT1/3/5	No
NSC 319726	N	99.9	99.8	99.9	25.3	28.0	2.0	p53	[32]
Tenovin-1	N	96.9	32.0	26.7	−1.2	5.0	−6.9		
RITA (NSC 652287)	N	83.6	50.2	43.9	16.8	23.3	−1.2		
Ciclopirox ethanolamine	N	100.0	99.8	99.9	9.0	0.4	−15.9	Iron chelator	No
Econazole nitrate (Spectazole)	Y	85.8	55.1	56.7	2.8	10.9	−2.8	Antifungal	No
Miconazole nitrate	Y	76.1	58.3	59.9	12.0	13.0	−2.3		
Monensin sodium salt (Coban)	N	92.2	96.7	96.6	24.3	34.4	7.2	Antibiotic	No
Idoxuridine	Y	82.3	85.8	85.3	−5.9	−3.9	0.4	Antiviral	[33]
3-Deazaneplanocin A (DZNeP)	N	97.4	76.8	75.7	1.0	42.5	−5.1	Purine analog	No
Fludarabine (Fludara)	Y	99.6	99.1	99.1	−9.4	10.8	−1.0		
Clofarabine	Y	100.0	99.8	100.0	−5.9	1.3	4.3		
Azaguanine-8	N	99.6	12.7	6.7	2.9	21.9	0.7		
Cytarabine	Y	99.5	99.8	100.0	2.1	13.3	0.0	Antimetabolic agent and DNA synthesis inhibitor	[34]
Gemcitabine	Y	100.0	99.9	100.0	7.7	9.0	6.8	Nucleoside analog	[35]
Cyclocytidine HCl	N	100.0	99.8	100.0	−0.6	17.2	7.2		
Trifluridine (Viroptic)	Y	100.0	99.9	100.0	−5.7	9.3	−3.1		
Azacitidine (Vidaza)	Y	98.8	99.9	100.0	9.2	7.1	3.8	DNA methylation	No
Floxuridine	Y	96.7	62.6	48.4	14.4	73.2	0.0	MDCK/PEPT1.	No
Abitrexate (Methotrexate)	Y	93.4	62.2	67.5	−12.6	56.4	1.3	antifolate antimetabolite	No
Triapine	N	100.0	99.9	100.0	9.9	14.2	−22.3	ribonucleotide reductase;	[36]
Vidarabine (Vira-A)	Y	88.1	99.7	100.0	−4.4	−4.3	5.6	adenosine with the D-ribose	[37]
Apigenin	N	89.8	54.8	53.7	17.4	32.1	5.6	Cytochrome P450	No
Avasimibe	N	63.3	50.9	47.8	7.1	19.6	3.6		
Cyclosporin A	N	73.2	67.2	46.8	27.4	24.5	−5.1		
ML130	N	70.6	35.9	23.4	6.9	13.6	−5.3	NOD1	No
PAC-1	N	95.1	95.5	95.0	14.3	16.5	−7.7	procaspase-3 activator	[38]
ABT-263 (Navitoclax)	N	64.1	33.1	73.9	−2.2	5.5	−7.6	BCL protein	[39,40]
ABT-737	N	72.1	26.6	55.7	4.7	16.3	−4.7		
PF-2545920	N	99.6	33.7	41.1	5.5	11.2	−1.0	PDE10A	No
Cladribine	Y	99.8	99.8	100.0	−8.8	−4.1	1.4	adenosine deaminase	[41,42]
Adefovir Dipivoxil (Preveon, Hepsera)	Y	100.0	99.8	100.0	9.7	8.1	−2.1	reverse transcriptase	No
TPCA-1	N	78.8	78.5	77.1	18.5	30.7	−3.7	IKK-2	[43]
Mycophenolic (Mycophenolate)	Y	98.3	96.7	97.1	27.7	35.3	−2.3	inosine monophosphate dehydrogenase	No
PTC-209	N	89.3	90.7	91.5	31.6	38.5	−2.3	BMI-1, polycomb complex protein	No
EX 527 (Selisistat)	N	71.7	30.5	29.6	17.2	41.4	0.0	Sirtuin 1 (SIRT1)	No
Salinomycin (Procoxacin)	N	95.9	92.5	96.8	19.5	35.1	−1.7	Aromatase	No
Dapivirine	N	70.1	41.7	34.2	37.3	70.3	8.3	Nonnucleoside reverse transcriptase	No
CCG 50014	N	85.0	35.8	30.2	5.7	2.4	5.8	RGS4, G-protein signaling 4	No
Mocetinostat (MGCD0103)		75.9	46.9	27.0	33.0	74.1	−0.3	HDAC	[44]
Atorvastatin calcium (Lipitor)	Y	65.7	25.4	30.6	10.2	42.3	−3.2	HMG-CoA reductase	No
UK 383367	N	73.7	17.0	33.6	17.0	29.4	−3.0	BMP-1 (C-proteinase) peptidase	No
Lonafarnib (SCH66336)	N	66.1	36.2	71.2	10.7	13.5	−5.9	FPTase, prenyltransferases	No
BX-912	N	68.0	42.3	45.6	34.3	55.5	0.3	PDK1: pyruvate dehydrogenase kinase 1	No
URB597	N	60.7	21.7	−9.2	−5.0	−3.7	−5.6	FAAH: fatty acid amide hydrolase	[45]
Anagrelide HCl	Y	97.4	58.4	52.9	34.1	89.3	−5.5	phosphodiesterase	No
Drospirenone	Y	98.0	57.4	56.3	31.7	87.3	8.9	Hormone	[46]
Ethinyl Estradiol	Y	98.0	53.3	44.3	42.9	89.0	0.8		
Norethindrone (Norethisterone)	N	98.8	48.4	58.4	42.2	90.8	−4.6		
Ganetespib (STA-9090)	N	88.8	99.7	100.0	32.9	28.6	−96.8	Heat shock protein 90 (HSP90)	[22]
PF-04929113 (SNX-5422)	N	95.4	99.4	99.7	33.0	26.2	1.0		
NMS-E973	N	96.0	99.1	99.3	33.8	21.5	−3.5		
XL888	N	95.5	99.6	99.7	38.7	38.9	−3.4		
17-AAG	N	97.3	99.4	99.5	39.9	50.9	10.0		
VER-49009	N	95.7	98.9	99.0	30.7	26.5	−8.9		
SNX2112	N	95.3	99.4	99.6	29.3	26.1	1.4		
BIIB021	N	87.0	99.8	99.9	34.5	28.6	−5.6		
AT13387	N	87.3	99.8	99.9	37.9	31.7	−68.5		
NVP-BEP800	N	87.6	98.8	98.6	28.7	13.2	−7.4		
AUY922 (NVP-AUY922)	N	89.5	99.8	100.0	34.6	40.8	−72.5		
KW-2478	N	81.9	92.7	92.7	33.7	25.7	−2.7		
Vincristine	Y	95.3	96.6	96.4	45.8	76.9	−7.2	Microtubules	[47,48,49,50]
ABT-751 (E7010)	N	74.3	81.5	82.7	41.4	74.5	−117.1		
Epothilone A	N	68.0	27.3	71.6	32.5	84.1	−3.4		
Amygdalin	N	73.3	58.3	53.9	4.7	11.7	−0.3	Natural product, Vit B17	No
Kaempferol	N	84.8	64.7	60.2	7.0	14.7	1.4	Natural product, flavonoid antioxidant	No
ENMD-2076	N	66.5	72.3	73.4	47.7	81.7	−7.3	Aurora A and B	No
AMG-900	N	69.7	50.4	45.4	3.5	15.4	−7.5		
MLN8054	N	80.1	36.2	30.7	−2.8	5.2	3.3		
PCI-32765 (Ibrutinib)	Y	99.7	74.9	82.8	6.9	−7.7	−2.4	Bruton’s tyrosine kinase (BTK),	No
CNX-774	N	90.1	70.0	62.8	39.6	60.3	3.4		
AR-A014418	N	97.0	47.9	46.5	0.4	17.8	0.6	GSK3β	No
CGP 57380	N	70.1	58.1	53.7	17.6	21.3	−2.4	MNK1, Ser-Thr PK	No
Genistein	N	88.6	40.6	27.0	−6.7	18.2	−4.9	protein tyrosine kinase (PTK)	No
HMN-214	N	77.0	58.9	64.6	20.0	84.6	1.7	Polo-like kinase (Plk)1	No
TG003	N	79.3	46.7	46.1	8.1	25.3	−1.9	Cdc2-like kinase (Clk)	No
Skepinone-L	N	60.7	47.1	39.9	−1.5	−3.0	5.9	MAPK	[51,52,53]
TAK-632	N	98.9	65.2	52.8	1.2	34.0	1.6		
YM201636	N	81.6	56.3	44.8	45.9	62.1	6.3	PIKfyve	[54]
MK-2461	N	67.0	57.0	56.5	30.1	41.8	−2.1	c-Met	[55]
OSI-930	N	90.6	43.5	26.5	8.3	15.1	10.1	Kit and KDR	[56]
SP600125	N	95.7	50.5	53.3	30.7	59.4	2.3	JNK	[57,58]
SKI II	N	72.2	65.1	63.4	13.2	29.0	−3.4	PI3K/mTOR (mammalian target of rapamycin)	[25,59,60,61,62]
PKI-402	N	74.1	44.9	50.4	43.1	51.8	−0.2		
BEZ235 (NVP-BEZ235)	N	72.9	37.4	32.7	33.7	17.7	−1.5		
WYE-125132	N	87.6	67.6	54.9	45.8	58.2	−13.0		
OSI-027	N	74.1	68.3	62.2	36.1	27.3	−1.0		
Torin 1	N	64.6	57.3	65.9	36.1	37.8	−5.0		
Ku-0063794	N	69.3	55.3	57.9	28.3	25.5	3.0		
AZD2014	N	74.8	70.3	73.3	30.6	36.3	−3.9		
Nocodazole	N	92.4	76.2	76.5	48.3	77.3	−3.0	Abl and src	[63,64]
Dasatinib (BMS-354825)	Y	64.7	74.2	84.5	20.3	45.9	−0.8		
KX2-391	N	93.5	65.1	84.4	34.7	78.3	−4.2		
Bosutinib (SKI-606)	Y	72.8	81.8	77.1	20.3	28.5	5.3		
PP1	N	84.3	35.8	37.8	37.6	56.3	−0.2		
AZ 960	N	84.1	82.1	82.4	45.5	77.6	1.4	Janus kinase-2 (JAK2)	No
LY2784544	N	71.9	65.7	67.0	25.5	74.6	−0.2		
WHI-P154	N	83.6	66.3	62.0	10.3	16.5	−2.7	JAK3	No
Cyt387	N	62.4	45.2	41.9	26.3	35.6	4.3	JAK1 and JAK2	No
KPT-276	N	88.8	27.6	11.3	25.2	43.0	9.2	Chromosomal maintenance 1 (CRM1)	[27]
KPT-185	N	79.3	26.2	18.9	33.4	68.0	0.5		
Phloretin	N	68.3	−27.0	−23.8	8.6	24.4	−3.9	Sodium/glucose cotransporter 1 and 2	No
Ivacaftor (VX-770)	Y	82.6	75.5	77.1	37.5	46.3	−3.3	CFTR, chloride channel	No
Tolbutamide	Y	99.8	99.9	100.0	62.0	81.2	−2.9	Potassium channel blocker	No
Fexofenadine HCl	Y	63.8	10.5	27.6	−3.0	−1.8	−6.4	histamine H1 receptor	No
Bergapten	N	76.4	7.6	10.4	14.4	36.1	−0.4	DNA crosslinks	No
SC144	N	99.9	98.4	98.6	15.7	17.7	−4.2	gp130, cytokine receptors	No
Daidzein	N	78.5	32.2	−9.2	7.8	22.2	−3.0	Peroxisome proliferator-activated receptor	No
CEP-18770 (Delanzomib)	N	99.5	99.7	99.8	30.9	94.6	−4.5	Proteasome	[28,65]
MLN9708	N	99.1	99.6	99.7	39.9	92.0	−1.9		
Oprozomib (ONX 0912)	N	99.8	99.5	99.6	40.7	96.4	−6.1		
MG-132	N	99.5	98.9	99.3	48.6	97.5	−2.7		
MLN2238	N	99.5	99.7	99.8	39.4	93.3	−3.2		
ONX-0914 (PR-957)	N	99.7	99.5	99.7	52.5	95.2	1.7		
Amonafide	N	98.5	99.3	99.8	30.5	43.8	−4.6	Topoisomerase II	[66]
Teniposide (Vumon)	Y	96.4	97.9	98.8	27.5	92.8	7.4		
Etoposide (VP-16)	Y	84.6	60.1	63.5	0.0	32.9	2.6		
Golvatinib (E7050)	N	70.4	79.8	72.2	28.9	32.3	−9.6	VEGFR and epidermal growth factor receptor (EGFR)	[23]
Sorafenib (Nexavar)	Y	78.5	72.6	89.4	23.1	45.1	−5.3		
Tyrphostin AG 1296 (AG 1296)	N	99.0	53.2	48.6	36.4	91.9	−19.3		
NVP-TAE226	N	93.1	91.5	90.7	41.5	68.2	−2.9		
Cabozantinib malate	Y	77.9	79.3	81.3	27.4	39.3	0.3		
XL-184 (Cabozantinib)	Y	50.4	64.2	64.6	−29.2	45.5	−1.4		
Linifanib (ABT-869)	N	79.5	60.5	55.6	20.7	35.3	−13.9		
Dovitinib (TKI-258) Dilactic Acid	N	97.3	67.6	57.9	11.8	32.8	8.2		
AZD4547	N	75.7	81.5	80.0	42.3	66.4	8.7		
AG-1024	N	91.6	71.8	66.4	5.4	9.7	17.0		
BMS-754807	N	90.0	61.8	53.2	43.6	61.5	9.3		
Afatinib (BIBW2992)	Y	89.2	95.2	94.2	34.8	69.9	−0.3		
Butein	N	92.3	62.6	60.1	42.7	64.3	−4.5		
CO-1686 (AVL-301)	N	96.7	74.3	76.9	45.5	67.9	−6.1		
PD168393	N	80.9	67.2	57.1	17.0	37.0	−5.8		
PD153035 HCl	N	72.2	74.7	65.4	13.9	14.2	−4.1		
Gefitinib (Iressa)	N	77.5	91.2	89.3	17.8	−10.2	−3.1		
Desmethyl Erlotinib (CP-473420)	Y	89.1	75.5	70.1	16.3	28.1	6.0		
OSI-420 (Desmethyl Erlotinib)	N	86.2	46.7	42.4	16.2	26.8	5.1		
Neratinib (HKI-272)	Y	84.7	56.7	55.6	10.4	25.8	0.3		
Erlotinib HCl	N	76.9	66.8	62.8	8.7	14.5	−0.6		
WZ8040	N	99.2	96.4	96.9	78.7	92.0	1.4		

**Table 2:** JAK-STAT3 inhibitors identified after two-round of screens.

Compound	Target
Niclosamide	STAT3
Nifuroxazide	STAT1/3/5
AZ 960	JAK2
LY2784544	JAK2
Cyt387	JAK1/2
Fludarabine	STAT1
WHI-P154	JAK3
SC144	IL-6 receptor
